# Selective small-molecule EPAC activators

**DOI:** 10.1042/BST20190254

**Published:** 2019-10-11

**Authors:** Urszula Luchowska-Stańska, David Morgan, Stephen J. Yarwood, Graeme Barker

**Affiliations:** 1 Institute of Biological Chemistry, Biophysics, and Bioengineering, Heriot-Watt University, Edinburgh EH14 4AS, U.K.; 2 Institute of Chemical Sciences, Heriot-Watt University, Edinburgh EH14 4AS, U.K.

**Keywords:** cAMP, exchange proteins, signalling

## Abstract

The cellular signalling enzymes, EPAC1 and EPAC2, have emerged as key intracellular sensors of the secondary messenger cyclic 3′,5′-adenosine monophosphate (cyclic adenosine monophosphate) alongside protein kinase A. Interest has been galvanised in recent years thanks to the emergence of these species as potential targets for new cardiovascular disease therapies, including vascular inflammation and insulin resistance in vascular endothelial cells. We herein summarise the current state-of-the-art in small-molecule EPAC activity modulators, including cyclic nucleotides, sulphonylureas, and *N*-acylsulphonamides.

## Introduction

Cyclic adenosine monophosphate (cAMP) is a prototypical secondary messenger involved in the regulation of many cellular processes in response to extracellular stimuli [1,2]. cAMP signalling is regulated by the relative expression and localisation of adenylyl cyclases (ACs) and phosphodiesterases (PDEs) within the cell [3–9]. cAMP mediates its effects mostly via protein kinase A (PKA) [10–13] and exchanges proteins activated by cAMP (EPACs) [14–16] in addition to Popeye domain-containing proteins (POPDCs) [17–20] and cyclic nucleotide-gated ion channels (CNGs) [21–24]. Controlling diverse physiological responses, many drugs have been developed to elevate intracellular cAMP levels, either through inhibition of PDEs or activation of ACs [7,25–34]. However, the indiscriminate nature of cAMP signalling points to the potential to concurrently activates all of the cAMP-sensor signalling pathways [35–38]. In this review, we summarise recent progress in the development of selective small-molecule activators of EPACs.

EPAC1 and EPAC2 are multi-domain proteins, encoded by different genes, which act as guanine nucleotide exchange factors for the Ras-like GTPases Rap1 and Rap2 [15,16,39]. They differ by function and tissue expression patterns, but share the same functional domain organisation and mode of activation. In each case, the structure consists of a regulatory *N*-terminal domain with a dishevelled-EGL pleckstrin homology domain and a cyclic nucleotide-binding domain (CNBD), and a *C*-terminal domain which includes a Ras exchange motif (REM), a Ras association domain and a CDC25 homology domain [40–44]. EPAC2 contains an additional *N*-terminal CNBD with a reduced affinity for cAMP, thought to be involved in subcellular localisation [15,16,45]. In the inactive state, the proteins exist in an auto-inhibited conformation in which the regulatory domain blocks the Rap-binding site [42,43,46]. Binding of cAMP to the CNBD induces a conformational shift, which unveils the Rap-binding site, allowing for signal transduction [42–44].

Many studies have suggested that EPAC signalling dysfunction plays a role in such diverse conditions as hypertension [47,48], diabetes [49], cancer [50,51], cardiac arrhythmia [52], and inflammatory pain [53]. EPAC1 has been shown to play a role in mitigating pro-inflammatory cytokine signalling in vascular endothelial cells (VECs), through induction of SOCS3 and subsequent inhibition of IL-6 signalling via the JAK/STAT3 pathway [54,55]. In the context of lung inflammation, EPAC1 and 2 appear to play disparate roles in the IL-8 signalling pathway associated with the chronic obstructive pulmonary disorder (COPD), in which EPAC1 suppresses airway remodelling, while EPAC2 is pro-inflammatory [56–60].

With involvement in varied disease states [14], it is unsurprising that EPAC activity modulators have attracted attention as both tool molecules for further elucidating EPAC signalling roles and as drug development candidates. With differing and often opposing roles played by EPAC1 and 2 as well as other cAMP sensors, any EPAC1 or 2 agonist or antagonist would ideally be specific for a given EPAC isoform. In the case of EPAC antagonists, a recent review [14] has highlighted the use of selective inhibitors in various disease models; including the uncompetitive (CE3F4) and non-competitive EPAC1 (5225554 and 5376753) inhibitors and the EPAC2-selective inhibitor, ESI-05 (4-methylphenyl-2,4,6-trimethylphenylsulphone). For example, CE3F4 has been shown to inhibit autophagy in cardiomyocytes [61] and cardiac arrhythmia [62] and ESI-05 attenuates brain injury and neurological impairment [63,64] and inhibits pancreatic cancer cell migration [65]. ESI-09 (3-[5-(tert-butyl)isoxazol-3-yl]-2-[2-(3-chlorophenyl)hydrazono]-3-oxopropanenitrile) has also been defined as a non-selective inhibitor of EPAC1 and EPAC2 [66]. Herein, we review recent progress in the development of selective EPAC activators.

## Cyclic nucleotide EPAC ligands

**Figure 1.**
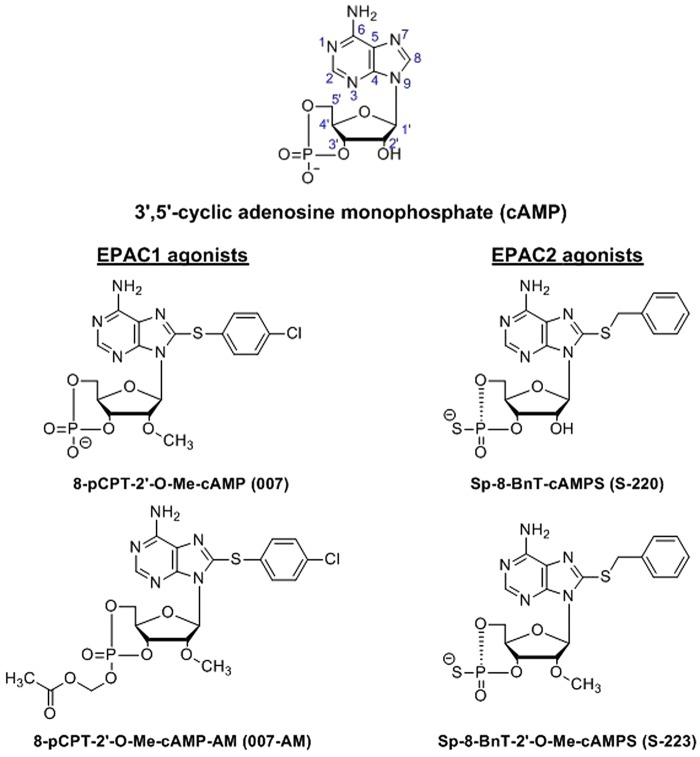
Extant cyclic nucleotide EPAC agonists. Natural EPAC agonist cAMP compared to the EPAC1-selective chlorothiophenyl analogue 007 and its acetoxymethyl ester 007-AM which displays increased cell permeability. Also shown are the EPAC2-selective thiobenzyl thiophosphates S-220 and S-223.

While cAMP activates both EPAC1 and EPAC2, many isoform-selective eight substituted cAMP analogues have been reported, the thioarylnucleotide 007 and its acetoxymethyl derivative 007-AM as well as the EPAC2-selective thiobenzyl thiophosphates S-220 and S-223 (Figure 1).

### 007 and 007-AM

Enserink et al. first developed the EPAC-selective cAMP analogue, 8-(4-chlorophenylthio)-2′-*O*-methyladenosine-3′,5′-cyclic monophosphate (8-pCPT-2′-O-Me-cAMP, 007) as a tool to discriminate the respective roles of EPAC and PKA signalling pathways [67]. The CNBDs of PKA and CNGs have conserved glutamic acid residues that form a hydrogen bond with the 2′-hydroxy group of the cAMP molecule [67,68], while CNBDs of both EPAC1 and EPAC2 have different corresponding amino acids, glutamine (Q270 and lysine (K405), respectively [67,69]. It was discovered that the 2′-hydroxy group of cAMP is not necessary for the binding and activation of EPAC1, but 2′-*O*-alkyl substitutions, such as 2′-O-methyl, 2′-O-ethyl, 2′-O-propyl or 2′-*O*-butyl in cAMP analogues, greatly reduce their affinity for PKA [67,70]. 007 was shown to not only be an EPAC-selective agonist, but also a more potent EPAC1 activator than cAMP (see Table 1) [67,69]. Further study demonstrated that the *p*-chlorophenylthio (pCPT) substituent at the eight positions is responsible for high affinity, which Schwede et al. attributed to hydrophobic interactions in the binding pocket and the chlorophenyl motif shielding the binding pocket against solvent [69]. 2′-*O*-methylation provides not only discrimination against all four PKA isoforms in *in vitro* kinase assays (see Table 2), but also high maximal activity, presumably caused by the 2′-*O*-Me group pushing away Q270, which then interacts with the hinge region and changes its conformation to a more favourable one. While 007 activates both EPAC isoforms in *in vitro* Rap1 activation assays, importantly it activates EPAC1 to a greater degree (*k*_max_ = 3.3 for EPAC1 vs. *k*_max_ = 0.8 for EPAC2) as well as binding EPAC1 more strongly (with AC_50_ = 1.8 µM and for EPAC1 compared with AC_50_ = 3.5 µM for EPAC2, see: Table 1), due to the single amino acid difference between their cAMP-binding sites, as detailed above [69].

**Table 1:** Comparison of activation constants for EPAC1 and EPAC2 obtained by *in vitro* biochemical Rap1 activation assays

	EPAC1		EPAC2	
	AC_50_ [µM]	*k*_max_	AC_50_ [µM]	*k*_max_
cAMP	45	1.0	1.8	1.0
007	1.8	3.3	3.5	0.8
S-220	13	0.3	0.1	7.7
S-223	30	0.2	1.5	4.7

Half-maximal concentration for activation (AC_50_) describes the affinity of EPAC isoform for the cyclic nucleotide. Relative maximal activity (*k*_max_) is the activity observed under saturating concentrations of the ligand and it is a measure of nucleotide's capability to shift the equilibrium towards the active state of EPAC.

**Table 2:** Comparison of apparent activation constants (*K*_act_) for four PKA isoforms obtained by *in vitro* biochemical kinase assay

	*K*_act_ [µM]			
	PKA-Iα	PKA-Iβ	PKA-IIα	PKA-IIβ
cAMP	0.085	0.038	0.080	0.19
007	14	18	>70	50
S-220	0.29	0.29	0.27	0.21
S-223	>1000	>1000	>25	>1000

**Scheme 1.**
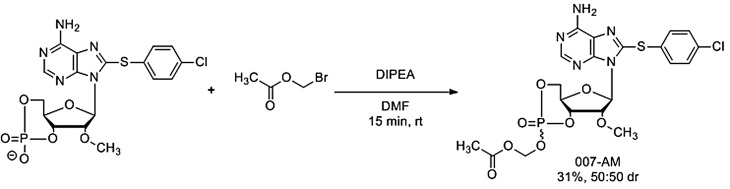
Synthesis of 007-AM from 007. 007-AM may be prepared from 007 by the reaction of bromomethyl acetate with 007 in the presence of diisopropylethylamine in dimethylformamide to give 007-AM in 31% yield and 50:50 dr after 15 min at room temperature.

A disadvantage of 007 is its poor membrane permeability due to the presence of the extremely polar-charged phosphate [71–73]. Vliem et al. have developed an acetoxymethyl ester of 007 (007-AM) to overcome this problem via a simple protection protocol with bromomethyl acetate to give the product in ∼50:50 diastereomeric ratio (dr) and 31% yield (Scheme 1) [71]. While the diastereomers were separable, the pharmacokinetics of both were shown to be similar, so the mixture was used in bioactivity assays. Masking the phosphate group with a labile ester improves cell membrane penetration and subsequent hydrolysis of the ester by water or cellular esterases releases the active molecule, a strategy first employed by Schultz et al. to improve the permeability of dibutyryl cAMP [73]. Detection of active Rap1 in cell lysates and a FRET-based cellular assay demonstrated that 007-AM activates EPAC1 more readily than 007, and can be used at concentrations two to three orders of magnitude lower to achieve comparable effect [71], indicating that membrane permeability was indeed significantly improved over the parent 007.

**Scheme 2.**
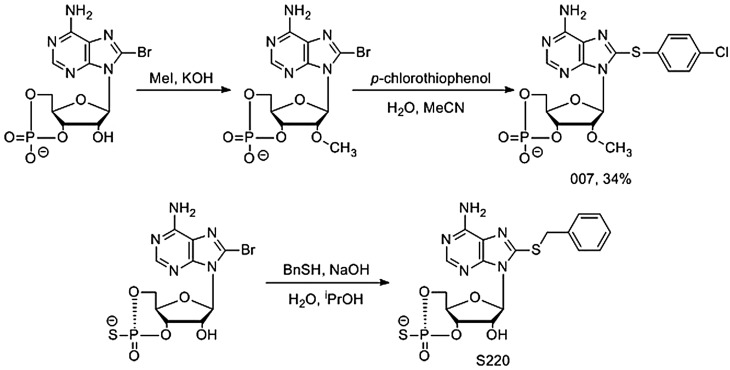
Synthesis of cyclic nucleotide EPAC activators. 007 is prepared from 8-bromo-cAMP *via* methylation with iodomethane followed by nucleophilic displacement of bromine using *p*-chlorothiophenol. In an analogous procedure, S-220 is prepared from 8-bromo-cAMPS *via* nucleophilic attack of benzyl thiol.

007 and its esterified analogue have been widely used for investigating EPAC1 signalling pathways as well as for *in vivo* experiments [54,74–82] where the administration of 007 was reported to decrease renal failure [83] and oxidative stress [84] in ischaemia-reperfusion injury model mice. While both compounds are useful tool molecules, their utility as drug development candidates is lacking; plausible analogue syntheses are largely limited to sugar protections and substitution in the adenosine eight-position (see Scheme 2). Additionally, EPAC1-selective 007 is also a weak EPAC2 agonist (see Table 1), leading to off-target effects. Hothi et al. reported that activation of EPAC proteins in cardiomyocytes with 007 is associated with disturbed calcium homeostasis and arrhythmia [85]. Further studies on cardiomyocytes isolated from wild-type (WT), EPAC1 knockout (EPAC1-KO) and EPAC2 knockout (EPAC2-KO) mice have shown that the Ca^2+^ leak observed in both WT and EPAC1-KO after treatment with 007 did not occur in EPAC2-KO, pointing to the EPAC2 isoform as a mediator of this effect [86].

**Figure 2.**
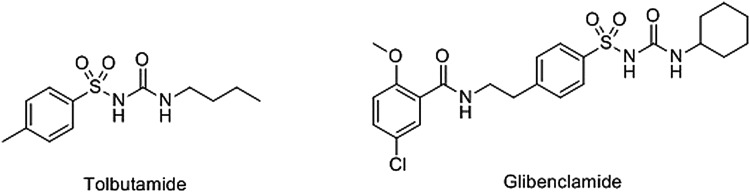
Two sulphonylurea drugs. Approved sulphonylurea drugs include the anti-diabetics tolbutamide and gilbenclamide.

### S-220 and S-223

The EPAC2-selective agonist 8-benzylthioadenosine-3′,5′-cyclic monophosphorothioate (Sp-8-BnT-cAMPS, S-220) was developed by Schwede et al. [69]. Improved maximal activity and affinity for EPAC2 were achieved by introducing a benzylthio (BnT) substituent at eight positions of adenine vs. the thioaryl present in the EPAC1-selective agonists 007 and 007-AM. Inspection of a crystal structure of S-220 bound to EPAC2 in the active conformation reveals that the BnT group is sandwiched between K450 (part of the lid, unique for EPAC2) and L379 (in the CNBD domain) and utilises hydrophobic interactions to promote the active conformation of CNBD. Replacing the axial oxygen atom of cyclic phosphate with sulfur provides an additional increase in maximal activity, while the crucial interaction with the phosphate-binding cassette (PBC) is retained [69]. Site-directed mutagenesis has shown that the single amino acid difference within cAMP-binding sites of EPAC1 and EPAC2 is responsible for S-220 isoform-selectivity (EPAC2 is activated with AC_50_ = 0.1 μM, while EPAC1 with AC_50_ = 13 μM, see Table 1). In *in vitro* kinase assays, S-220 was shown to activate all four PKA isoforms, though to a lesser extent than cAMP (see Table 2). In cell-based studies utilising the U2OS cell line stably transfected to overexpress EPAC2, however, only very low PKA activation was observed suggesting that this may not be a problem *in vivo* [69]. 2′-substitutions, which in the case of 007 resulted in highly improved selectivity for EPAC1 over PKA [67,69], considerably reduce the maximal activity and affinity for EPAC2, as in 2′-*O*-methylated S-220 analogue, S-223 (*k*_max_ = 4.7 and AC_50_ = 1.5 μM vs. *k*_max_ = 7.7 and AC_50_ = 0.1 for S-223 and parent compound, respectively). Although efficiently discriminating against PKA in *in vitro* kinase assays (see Table 2) and demonstrating reduced, but still significant potency for EPAC2 activation, S-223 failed to induce EPAC2 activity in cell-based tests [69]. S-220 was reported to enhance glucose-induced insulin release from isolated primary human islets [69]. It was also used for *in vivo* studies, where mice on high-fat diet treated with S-220 displayed reduced body weight gain [87].

### Synthesis of cyclic nucleotide EPAC agonists

Both 007 and S-220 are accessed via bromonucleotide precursors (Scheme 2). For example, 8-bormo-cAMP may be efficiently methylated in the 2′-position using iodomethane, and subsequent treatment with 4-chlorothiophenol gives 007 in 34% yield [69]. Following an analogous procedure, S-220 may be synthesised via the treatment of 8-bromo-cAMPS with benzyl mercaptan (Scheme 2) — synthetic yields were not reported by the authors [69]. While both 8-bromo-cAMP and 8-bromo-cAMPS are commercially available, substitutions in other positions are synthetically challenging, limiting the potential of cyclic nucleotide EPAC ligands as drug development candidates.

## Non-nucleotide EPAC ligands

Two classes of non-cyclic nucleotide small-molecule EPAC activators have emerged in recent years; sulphonylureas (SUs) are one such class. These compounds were initially of interest for use as anti-diabetic drugs, with clinically approved examples including Tolbutamide (TLB) and Glibenclamide (GLB) (Figure 2). SUs stimulates the secretion of insulin by closing pancreatic β cell *K*_ATP_ channels through binding to sulphonylurea receptor 1 (SUR1) [88].

Evidence for SU activity at EPAC1 and 2 has been controversial — Sunaga et al. have previously reported that SUs are selective activators of the EPAC2 isoform using in-cell FRET biosensor imaging to screen for EPAC2 activation, further noting that SU-induced insulin secretion was reduced (though not eliminated) in mice lacking EPAC2 [88]. Using the same technique, Zhang et al. later confirmed direct binding of SUs to EPAC2, and that this binding was at an allosteric site [89]. Further investigation of an L408W point mutant (known to bind cAMP but with reduced Rap1 activation activity in the L273W EPAC1 homologue) [44,90] suggested that EPAC2 activation by SUs occurred via the same molecular mechanism as cAMP activation [89].

Recent studies by Tsalkova et al. dispute these findings, however, showing that SUs were unable to bind to or activate EPAC2 *in vitro* [91]. In a fluorescence-based competition assay where Rap—1-bound fluorescent Mant-GDP is exchanged with GDP in the presence of activated EPAC2 leading to a decrease in fluorescence, it was observed that GLB failed to activate EPAC2 in concentrations from 0.001 to 100 µM, whilst 300 µM led to robust Mant-GDP dissociation. The authors proposed that the supposed activation of EPAC2 may be due to the previously observed increase in cellular cAMP levels in the presence of Sus [92,93]. This had, however, been ruled out by Sunaga et al. in their original report and the mechanism of EPAC2 activation by SUs remains ambiguous. However, SUs have found a role as probe molecules for studying EPAC2-mediated cellular processes, for example, insulin exocytosis as reported by Barg et al. [94].

**Scheme 3.**
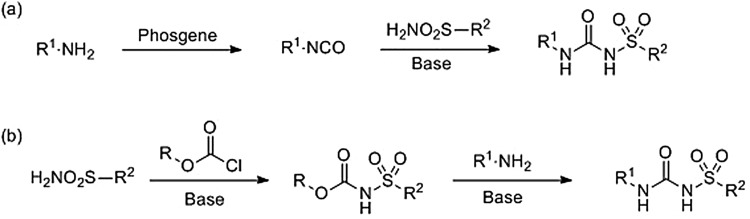
Sulphonyl urea synthesis. Sulphonyl ureas are commonly synthesised *via* one of the two routes: (**a**) from the primary amines by the phosgene-mediated formation of an isocyanate followed by nucleophilic attack of a primary sulphonamide or (**b**) reaction of a primary sulphonamide with a chloroformate then nucleophilic substitution using a primary amine

The ease of synthesis and existence of multiple synthetic route to SUs is an attractive quality for the design of analogues [95]. The precursor amines, sulphonamides, and chloroformate esters are either inexpensive or easy to prepare. Commonly, SUs may be accessed by the treatment of a starting amine with phosgene followed by nucleophilic attack by a primary sulphonamide onto the resultant isocyanate (Scheme 3, route (a)) [96,97]. Alternatively, toxic phosgene may be avoided by the reaction of a primary sulphonamide with a chloroformate or anhydride to form the corresponding carbamate, followed by treatment with an amine to yield the sulphonylurea (Scheme 3, route (b)) [97,98].

**Figure 3.**
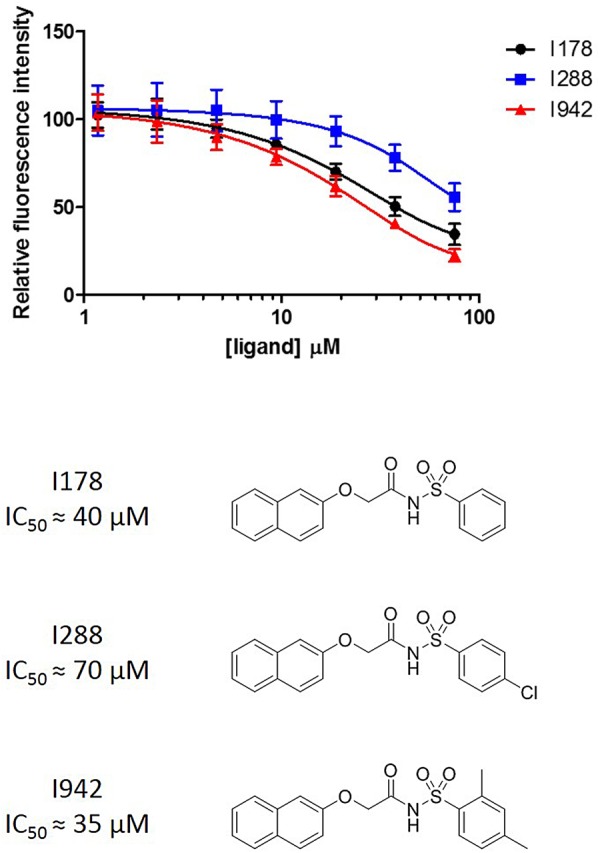
Dose–response curves for HTS hits against EPAC1-CNBD. The three hit compounds I178, I288, and I942 were tested in a seven-point dose–response curve in the EPAC1-CNBD, 8-NBD-cAMP-binding assay.

### *N*-acylsulphonamide EPAC agonists

Recently, Yarwood and co-workers reported the results of a screen of 5195 small molecules for binding at the EPAC1 CNBD, based on the competition for binding with the fluorescent cAMP analogue 8-NBD-cAMP [99]. The lead hit from the assay, I942, was found to have an IC_50_ of 35 µM compared with an IC_50_ of 4 µM for cAMP under the same conditions (Figure 3, alongside two other hit compounds from the same screen). Subsequent ligand-observed NMR studies confirmed the direct interaction of I942 with the CNBDs of both EPAC1 and EPAC2. The group then investigated EPAC activation by I942, based on activated EPAC-stimulated dissociation of fluorescent Mant-GDP from Rap1 in the presence of EPAC1/EPAC2 and I942, and observed partial agonist activity toward EPAC1, with very little concomitant activity at EPAC2. Notably, with I942 and cAMP binding the EPAC1 CNBD with roughly equal efficiency, the maximum activity induced by I942 is ∼10% that of cAMP. Rounding off the study, it was also shown that I942 had no effect *in vitro* on PKA activity, as measured by phosphorylation of a PKA substrate peptide [99]. Importantly, recent work from the Yarwood laboratory has demonstrated that I942 can activate Rap1 in cells overexpressing EPAC1, but not EPAC2 (manuscript in preparation). This precludes any non-specific action of I942 through inhibition of endogenous PDEs or activation of ACs.

The same group subsequently investigated the in-cell activity of I942 and were able to demonstrate EPAC1 and Rap1 activation in HEK293 T cells as well as SOCS3 induction and suppression of IL6-stimulated JAK/STAT3 signalling in HuVECs [54]. SOCS3 induction was blocked by the EPAC1 antagonists ESI-09 and EPAC1 siRNA, but not the PKA inhibitor H89, demonstrating that SOCS3 induction by I942 does indeed proceed via EPAC1. RNA sequencing identified 425 genes regulated by I942 in HuVECs, the same regulated by the EPAC1-selective cyclic nucleotide agonist 007 as well as forskolin (a common cAMP-elevating tool molecule) [100] and rolipram (a cAMP-elevating PDE-4 inhibitor) [101]. Finally, I942 was shown to block the expression of the cell adhesion molecule VCAM1, known to play a role in the development of cardiovascular inflammation [102]. While the use of I942 as a probe molecule is in its infancy, a recent study has reported the use of I942 as an EPAC1-specific activator renders VECs more susceptible to infection by Ebola virus [103].

**Figure 4.**
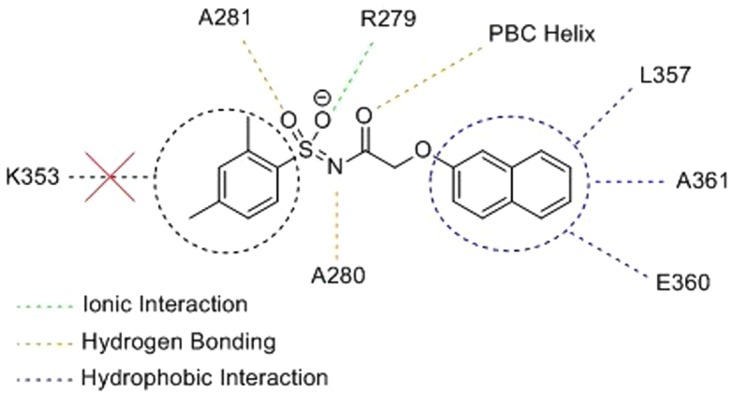
Proposed I942-binding interactions. Specific ligand-residue interactions from a computed docking model for I942 at an EPAC1 homology model cAMP-binding site. Note that I942 does not exploit interaction with K353, a key cAMP-EPAC1-binding moiety.

The mode of I942 binding at EPAC1 has yet to be fully elucidated, though we have advanced putative-binding models based on computational studies (Figure 4). In the absence of an EPAC1 structure, a homology model was constructed from an EPAC2 K405Q point mutant in the nucleotide-bound active conformation [55,69]. The findings suggest that the acidic *N*-acylsulphonamide moiety occupies the same volume as the cAMP phosphate, exploiting a key ionic interaction with R279 within the CNBD as well an engaging with charge-stabilised hydrogen bonds to A280 and A281; these residues are preserved in EPAC1. The model also suggests that the *m*-xylyl group of I942 occupies a similar space to that of the purine bicyclic ring of cAMP. However, I942 is unable to exploit the polar interactions available to cAMP through the adenine N1 and K353 on the REM domain *α*1 helix; this is proposed to be the key interaction, which stabilises the EPAC1 activation conformational reorganisation [42].

As an alternative EPAC1 activation model, we have proposed that, I942 may have access to some hydrophobic interactions through the naphthoxy group, which are not available to native cAMP. The model postulates that the oxymethylene motif threads a small passage, which leads to a hydrophobic channel that is occupied by the naphthoxy group of I942. Three residues L357, A361, and E360 on the REM *α*1 helix are theorised to stabilise the active state of EPAC1, and account for the agonistic properties of I942. The isoform-selectivity of I942 has been suggested to be due to the replacement of L357 and A361 by histidine and threonine, respectively, in EPAC2. The loss of L357 results in reduced surface contact with the naphthoxy group, whilst threonine induces steric hindrance. Furthermore, it was noted that packing of the naphthyl group against L357, A361, and E360 may stabilise the EPAC1 active state less effectively that cAMP, perhaps due to slightly altered seating of the CNBD against the core. Comparison of the three extant EPAC2 structures reveals a degree of plasticity in the positioning of the REM *α*1 helix and our docking models suggest a sterically crowded volume at the naphthyl–helix interface, accounting for the partial agonism of I942 due to this altered seating [55].

**Scheme 4.**
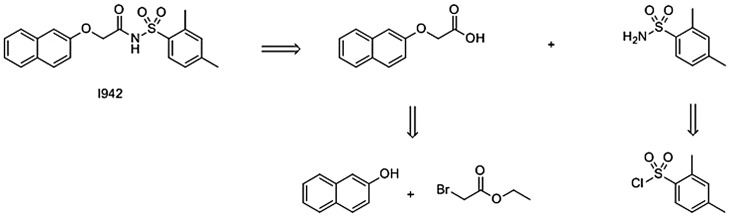
Retrosynthetic approach to I942. I942 may be disconnected *via* an amide formation to a primary sulphonamide (itself readily obtained from the corresponding sulfonyl chloride) and a naphthoxy acid formed from 2-naphthol and ethyl bromoacetate.

*N*-acylsulphonamides are common in modern medicinal chemistry; their synthesis and therapeutic potential have been recently reviewed [95]. In addition to phosphate mimetics, they have found use as carboxylate bioisosteres [104,105], with a p*K*_a_ in the range 3.5–4.5, similar to carboxylic acids [95]. An attractive feature of *N*-acylsulphonamides to the medicinal chemist is their ease of synthesis, with multiple synthetic routes starting from the readily obtained substrates [95]. For example, I942 may be disconnected (Scheme 4) back to a sulphonyl chloride, of which a SciFinder search reveals 16 532 distinct commercially available examples and phenol (13 674commercially available examples). This ease of synthesis and plurality of substrates for analogue synthesis furnishes *N*-acylsulphonamides and SUs with a significant advantage over cyclic nucleotide EPAC ligands as drug development hit compounds.

**Perspectives**

- Exchange proteins directly activated by cAMP (EPACs) have been shown to play an important role in the development of many diseases. The naturally occurring EPAC activator, cAMP, indiscriminately activates EPAC1 and EPAC2 as well as other cAMP sensors such as PKA and POPDCs. With different and often opposing biological activity originating from these species, drugs or probe molecules targeting EPAC1 or 2 would ideally do so selectively.
- Extant small-molecule EPAC activators include cAMP-mimetic cyclic nucleotides such as 007 and its more cell-permeable acetoxymethyl ester 007-AM (EPAC1 selective) as well as S-220 and S-223 (EPAC2 selective). While these examples have found diverse uses as probe molecules, issues remain around debatable selectivity (cyclic nucleotides) and cell permeability (007). Moreover, EPAC-specific cAMP analogues, as well as their cellular metabolites, have been reported to exert off-target effects [106–110]. Other examples include sulphonylurea EPAC2 activators and *N*-acylsulphonamide EPAC1 activators. However, SUs have an ambiguous mode of action and may not target EPAC2 in cells. For example, SUs do not increase insulin secretion in islets from SUR1 knockout mice, indicating that they may not be working through EPAC2 directly [111] and may involve and alternative pathway through interactions with β-arrestin [112].
- Recently identified *N*-acylsulphonamides provide a selective EPAC1 activator with a confirmed mode of activity *in vitro* and have demonstrated activity in cell and tissue cultures. With greater scope for analogue synthesis than cyclic nucleotides, we anticipate that future research in EPAC1 activators will focus on this compound series. The partial agonism displayed by I942 remains problematic in terms of therapeutic use due to competition with endogenous cyclic AMP as previously discussed [99]; however, it should be noted that I942 exerts agonist properties *in cellulae* [54], even in the presence of maximal cyclic AMP levels, as stimulated by a combination of forskolin and rolipram. Moreover, it can be anticipated that future I942 analogues may be designed to exploit additional binding/activation inducing interactions (e.g. through K353 via carboxylates of heterocylces) or to pose less of a steric challenge at the CNBD-REM *α*1 helix interface. Increasing potency of this series to full agonism will address much of the concern surrounding this compound series in the context of drug development.

## Abbreviations

AC: adenylyl cyclase
BnT: benzylthio
cAMP: cyclic adenosine monophosphate
CNBD: cyclic nucleotide-binding domain
CNGs: cyclic nucleotide-gated ion channels
COPD: chronic obstructive pulmonary disorder
dr: diastereomeric ratio
EPAC: exchange protein directly activated by cAMP
FRET: fluorescence resonance energy transfer
GDP: guanosine diphosphate
GLB: glibenamide
HTS: high-throughput screen
HuVECs: human vascular endothelial cells
IL-6: interleukin 6
KO: knockout
NMR: nuclear magnetic resonance
PBC: phosphate-binding cassette
*p*-CPT: *para*-chlorophenylthio
PDEs: phosphodiesterases
PKA: protein kinase A
POPDC: popeye domain-containing protein
REM: Ras exchange motif
SOCS3: suppressor of cytokine signalling 3
STAT3: signal transducer and activator of transcription 3
SU: sulphonylurea
SUR1: sulfonylurea receptor 1
TLB: tolbutamide
VCAM: vascular cell adhesion molecule
VEC: vascular endothelial cell
WT: wild type

## References

[1] Brown L.M., Rogers K.E., Aroonsakool N., McCammon J.A. and Insel P.A. (2014) Allosteric inhibition of Epac: computational modeling and experimental validation to identify allosteric sites and inhibitors. J. Biol. Chem. 289, 29148–29157 10.1074/jbc.M114.569319 25183009 PMC4200268

[2] Courilleau D., Bisserier M., Jullian J.-C., Lucas A., Bouyssou P., Fischmeister R. et al. (2012) Identification of a tetrahydroquinoline analog as a pharmacological inhibitor of the cAMP-binding protein Epac. J. Biol. Chem. 287, 44192–44202 10.1074/jbc.M112.422956 23139415 PMC3531735

[3] Dessauer C.W., Watts V.J., Ostrom R.S., Conti M., Dove S. and Seifert R. (2017) International union of basic and clinical pharmacology. CI. Structures and small molecule modulators of mammalian adenylyl cyclases. Pharmacol. Rev. 69, 93–139 10.1124/pr.116.013078 28255005 PMC5394921

[4] Halls M.L. and Cooper D.M.F. (2017) Adenylyl cyclase signalling complexes – pharmacological challenges and opportunities. Pharmacol. Ther. 172, 171–180 10.1016/j.pharmthera.2017.01.001 28132906

[5] Nicol X. and Gaspar P. (2014) Routes to cAMP: shaping neuronal connectivity with distinct adenylate cyclases. Eur. J. Neurosci. 39, 1742–1751 10.1111/ejn.12543 24628976

[6] Klussmann E. (2016) Protein–protein interactions of PDE4 family members — functions, interactions and therapeutic value. Cell. Signal. 28, 713–718 10.1016/j.cellsig.2015.10.005 26498857

[7] Maurice D.H., Ke H., Ahmad F., Wang Y., Chung J. and Manganiello V.C. (2014) Advances in targeting cyclic nucleotide phosphodiesterases. Nat. Rev. Drug Discov. 13, 290–314 10.1038/nrd4228 24687066 PMC4155750

[8] Maurice D.H., Wilson L.S., Rampersad S.N., Hubert F., Truong T., Kaczmarek M. et al. (2014) Cyclic nucleotide phosphodiesterases (PDEs): coincidence detectors acting to spatially and temporally integrate cyclic nucleotide and non-cyclic nucleotide signals. Biochem. Soc. Trans. 42, 250–256 10.1042/BST20130268 24646226

[9] Bassler J., Schultz J.E. and Lupas A.N. (2018) Adenylate cyclases: receivers, transducers, and generators of signals. Cell. Signal. 46, 135–144 10.1016/j.cellsig.2018.03.002 29563061

[10] Torres-Quesada O., Mayrhofer J.E. and Stefan E. (2017) The many faces of compartmentalized PKA signalosomes. Cell. Signal. 37, 1–11 10.1016/j.cellsig.2017.05.012 28528970

[11] Adame-García S.R., Cervantes-Villagrana R.D., Orduña-Castillo L.B., del Rio J.C., Gutkind J.S., Reyes-Cruz G. et al. (2019) cAMP-dependent activation of the Rac guanine exchange factor P-REX1 by type I protein kinase A (PKA) regulatory subunits. J. Biol. Chem. 294, 2232–2246 10.1074/jbc.RA118.006691 30530493 PMC6378977

[12] Calejo A. and Taskén K. (2015) Targeting protein–protein interactions in complexes organized by A kinase anchoring proteins. Front. Pharmacol. 6, 192 10.3389/fphar.2015.00192 26441649 PMC4562273

[13] Dema A., Perets E., Schulz M.S., Deák V.A. and Klussmann E. (2015) Pharmacological targeting of AKAP-directed compartmentalized cAMP signalling. Cell. Signal. 27, 2474–2487 10.1016/j.cellsig.2015.09.008 26386412

[14] Robichaux W.G. III and Cheng X. (2018) Intracellular cAMP sensor EPAC: physiology, pathophysiology, and therapeutics development. Physiol. Rev. 98, 919–1053 10.1152/physrev.00025.2017 29537337 PMC6050347

[15] Kawasaki H., Springett G.M., Mochizuki N., Toki S., Nakaya M., Matsuda M. et al. (1998) A family of cAMP-binding proteins that directly activate Rap1. Science 282, 2275–2279 10.1126/science.282.5397.2275 9856955

[16] de Rooij J., Zwartkruis F.J.T., Verheijen M.H.G., Cool R.H., Nijman S.M.B., Wittinghofer A. et al. (1998) Epac is a Rap1 guanine-nucleotide-exchange factor directly activated by cyclic AMP. Nature 396, 474–477 10.1038/24884 9853756

[17] Schindler R.F.R. and Brand T. (2016) The Popeye domain containing protein family – a novel class of cAMP effectors with important functions in multiple tissues. Prog. Biophys. Mol. Biol. 120, 28–36 10.1016/j.pbiomolbio.2016.01.001 26772438 PMC4821176

[18] Amunjela J.N. and Tucker S.J. (2017) Dysregulation of POPDC1 promotes breast cancer cell migration and proliferation. Biosci. Rep. 37, BSR20171039 10.1042/BSR20171039 28954821 PMC5696453

[19] Brand T. and Schindler R. (2017) New kids on the block: the Popeye domain containing (POPDC) protein family acting as a novel class of cAMP effector proteins in striated muscle. Cell. Signal. 40, 156–165 10.1016/j.cellsig.2017.09.015 28939104 PMC6562197

[20] Amunjela J.N. and Tucker S.J. (2016) POPDC proteins as potential novel therapeutic targets in cancer. Drug Discov. Today 21, 1920–1927 10.1016/j.drudis.2016.07.011 27458118

[21] Chen Z., Sun T. and Qing G. (2019) cAMP-modulated biomimetic ionic nanochannels based on a smart polymer. J. Mater. Chem. B 7, 3710–3715 10.1039/C9TB00639G

[22] Biel M. and Michalakis S. (2009) Cyclic nucleotide-gated channels In cGMP: Generators, Effectors and Therapeutic Implications (Schmidt H. H. H. W., Hofmann F. and Stasch J.-P., eds), pp. 111–136, Springer Berlin Heidelberg, Berlin, Heidelberg

[23] Michalakis S., Becirovic E. and Biel M. (2018) Retinal cyclic nucleotide-gated channels: from pathophysiology to therapy. Int. J. Mol. Sci. 19, 749 10.3390/ijms19030749 PMC5877610 29518895

[24] Podda M.V. and Grassi C. (2014) New perspectives in cyclic nucleotide-mediated functions in the CNS: the emerging role of cyclic nucleotide-gated (CNG) channels. Pflügers Arch. 466, 1241–1257 10.1007/s00424-013-1373-2 24142069

[25] Zuo H., Cattani-Cavalieri I., Musheshe N., Nikolaev V.O. and Schmidt M. (2019) Phosphodiesterases as therapeutic targets for respiratory diseases. Pharmacol. Ther. 197, 225–242 10.1016/j.pharmthera.2019.02.002 30759374

[26] Ammon H.P.T. and Müller A.B. (1985) Forskolin: from an ayurvedic remedy to a modern agent. Planta Med. 51, 473–477 10.1055/s-2007-969566 17345261

[27] Chen J., Hammell D.C., Spry M., D'Orazio J.A. and Stinchcomb A.L. (2009) In vitro skin diffusion study of pure forskolin versus a forskolin-containing *Plectranthus barbatus* root extract. J. Nat. Prod. 72, 769–771 10.1021/np800541k 19281221 PMC5082746

[28] Ahmad F., Murata T., Shimizu K., Degerman E., Maurice D. and Manganiello V. (2015) Cyclic nucleotide phosphodiesterases: important signaling modulators and therapeutic targets. Oral Dis. 21, e25–e50 10.1111/odi.12275 25056711 PMC4275405

[29] Southworth T., Kaur M., Hodgson L., Facchinetti F., Villetti G., Civelli M. et al. (2019) Anti-inflammatory effects of the phosphodiesterase type 4 inhibitor CHF6001 on bronchoalveolar lavage lymphocytes from asthma patients. Cytokine 113, 68–73 10.1016/j.cyto.2018.06.007 29934047

[30] Peng T., Gong J., Jin Y., Zhou Y., Tong R., Wei X. et al. (2018) Inhibitors of phosphodiesterase as cancer therapeutics. Eur. J. Med. Chem. 150, 742–756 10.1016/j.ejmech.2018.03.046 29574203

[31] Kim S.-H., Choi J., Lee K. and No K.T. (2017) Comparison of three-dimensional ligand-based pharmacophores among 11 phosphodiesterases (PDE 1 to PDE 11) pharmacophores. Bull. Korean Chem. Soc. 38, 1033–1037 10.1002/bkcs.11214

[32] Schwenkgrub J., Zaremba M., Joniec-Maciejak I., Cudna A., Mirowska-Guzel D. and Kurkowska-Jastrzębska I. (2017) The phosphodiesterase inhibitor, ibudilast, attenuates neuroinflammation in the MPTP model of Parkinson's disease. PLoS One. 12, e0182019 10.1371/journal.pone.0182019 28753652 PMC5533435

[33] Long T., Rojo-Arreola L., Shi D., El-Sakkary N., Jarnagin K., Rock F. et al. (2017) Phenotypic, chemical and functional characterization of cyclic nucleotide phosphodiesterase 4 (PDE4) as a potential anthelmintic drug target. PLoS Negl. Trop. Dis. 11, e0005680 10.1371/journal.pntd.0005680 28704396 PMC5526615

[34] Spadaccini M., D'Alessio S., Peyrin-Biroulet L. and Danese S. (2017) Pde4 inhibition and inflammatory bowel disease: a novel therapeutic avenue. Int. J. Mol. Sci. 18, 1276 10.3390/ijms18061276 PMC5486098 28617319

[35] Musheshe N., Schmidt M. and Zaccolo M. (2018) cAMP: from long-range second messenger to nanodomain signalling. Trends Pharmacol. Sci. 39, 209–222 10.1016/j.tips.2017.11.006 29289379

[36] Huang Y.Y., Xu M.X., Zhuang P.W. and Zhang Y.J. (2017) Advances on the Epac signal molecule in cardiovascular diseases. Chin. J N. Drugs 26, 2034–2039

[37] Cheng X., Ji Z., Tsalkova T. and Mei F. (2008) Epac and PKA: a tale of two intracellular cAMP receptors. Acta Biochimica Biophys. Sin. 40, 651–662 10.1111/j.1745-7270.2008.00438.x PMC2630796 18604457

[38] Schlepper M., Thormann J. and Mitrovic V. (1989) Cardiovascular effects of forskolin and phosphodiesterase-III inhibitors. Basic Res. Cardiol. 84, 197–212 10.1007/BF02650360 2530974

[39] Wang J.-C., Geng Y., Han Y., Luo H.-N. and Zhang Y.-S. (2018) Dynamic expression of Epac and Rap1 in mouse oocytes and preimplantation embryos. Exp. Ther. Med. 16, 523–528 10.3892/etm.2018.6253 30116310 PMC6090281

[40] Hoivik E.A., Witsoe S.L., Bergheim I.R., Xu Y., Jakobsson I., Tengholm A. et al. (2013) DNA methylation of alternative promoters directs tissue specific expression of Epac2 isoforms. PLoS One. 8, e67925 10.1371/journal.pone.0067925 23861833 PMC3701594

[41] Sugawara K., Shibasaki T., Takahashi H. and Seino S. (2016) Structure and functional roles of Epac2 (Rapgef4). Gene 575, 577–583 10.1016/j.gene.2015.09.029 26390815 PMC6636354

[42] Rehmann H., Arias-Palomo E., Hadders M.A., Schwede F., Llorca O. and Bos J.L. (2008) Structure of Epac2 in complex with a cyclic AMP analogue and RAP1B. Nature 27, 27 10.1038/nature07187 18660803

[43] Rehmann H., Das J., Knipscheer P., Wittinghofer A. and Bos J.L. (2006) Structure of the cyclic-AMP-responsive exchange factor Epac2 in its auto-inhibited state. Nature 439, 625–628 10.1038/nature04468 16452984

[44] Rehmann H., Prakash B., Wolf E., Rueppel A., de Rooij J., Bos J.L. et al. (2003) Structure and regulation of the cAMP-binding domains of Epac2. Nat. Struct. Biol. 10, 26–32 10.1038/nsb878 12469113

[45] Alenkvist I., Gandasi N.R., Barg S. and Tengholm A. (2017) Recruitment of Epac2A to insulin granule docking sites regulates priming for exocytosis. Diabetes 66, 2610–2622 10.2337/db17-0050 28679628

[46] Banerjee U. and Cheng X. (2015) Exchange protein directly activated by cAMP encoded by the mammalian rapgef3 gene: structure, function and therapeutics. Gene 570, 157–167 10.1016/j.gene.2015.06.063 26119090 PMC4556420

[47] Lakshmikanthan S., Zieba B.J., Ge Z.-D., Momotani K., Zheng X., Lund H. et al. (2014) Rap1b in smooth muscle and endothelium is required for maintenance of vascular tone and normal blood pressure. Arterioscler. Thromb. Vasc. Biol. 34, 1486–1494 10.1161/ATVBAHA.114.303678 24790136 PMC4224284

[48] Yu X., Zhang Q., Zhao Y., Schwarz B.J., Stallone J.N., Heaps C.L. et al. (2017) Activation of G protein-coupled estrogen receptor 1 induces coronary artery relaxation via Epac/Rap1-mediated inhibition of RhoA/Rho kinase pathway in parallel with PKA. PLoS One 12, e0173085 10.1371/journal.pone.0173085 28278256 PMC5344336

[49] Komai A.M., Musovic S., Peris E., Alrifaiy A., El Hachmane M.F., Johansson M. et al. (2016) White adipocyte adiponectin exocytosis is stimulated via β_3_-adrenergic signaling and activation of epac1: catecholamine resistance in obesity and type 2 diabetes. Diabetes 65, 3301–3313 10.2337/db15-1597 27554468

[50] Sun D.-P., Fang C.-L., Chen H.-K., Wen K.-S., Hseu Y.-C., Hung S.-T. et al. (2017) EPAC1 overexpression is a prognostic marker and its inhibition shows promising therapeutic potential for gastric cancer. Oncol. Rep. 37, 1953–1960 10.3892/or.2017.5442 28260059 PMC5367365

[51] Kong X., Ai G., Wang D., Chen R., Guo D., Yao Y. et al. (2019) PDE4 and Epac1 synergistically promote rectal carcinoma via the cAMP pathway. Anal. Cell. Pathol. 2019, 1–5 10.1155/2019/7145198 PMC6364102 30809467

[52] Yang Z., Kirton H.M., Al-Owais M., Thireau J., Richard S., Peers C. et al. (2017) Epac2-Rap1 signaling regulates reactive oxygen species production and susceptibility to cardiac arrhythmias. Antioxid. Redox Signal. 27, 117–132 10.1089/ars.2015.6485 27649969 PMC5510674

[53] Singhmar P., Huo X.J., Eijkelkamp N., Berciano S.R., Baameur F., Mei F.C. et al. (2016) Critical role for Epac1 in inflammatory pain controlled by GRK2-mediated phosphorylation of Epac1. Proc. Natl Acad. Sci. U.S.A. 113, 3036–3041 10.1073/pnas.1516036113 26929333 PMC4801297

[54] Wiejak J., van Basten B., Luchowska-Stańska U., Hamilton G. and Yarwood S.J. (2019) The novel exchange protein activated by cyclic AMP 1 (EPAC1) agonist, I942, regulates inflammatory gene expression in human umbilical vascular endothelial cells (HUVECs). Biochim. Biophys. Acta Mol. Cell Res. 1866, 264–276 10.1016/j.bbamcr.2018.11.004 30414891 PMC6325792

[55] Barker G., Parnell E., Van Basten B., Buist H., Adams D.R. and Yarwood S.J. (2017) The potential of a novel class of EPAC-selective agonists to combat cardiovascular inflammation. J. Cardiovasc. Dev. Dis. 4, 22 10.3390/jcdd4040022 PMC5753123 29367551

[56] Dekkers B.G.J., Racké K. and Schmidt M. (2013) Distinct PKA and Epac compartmentalization in airway function and plasticity. Pharmacol. Ther. 137, 248–265 10.1016/j.pharmthera.2012.10.006 23089371

[57] Zuo H., Cattani-Cavalieri I., Valença S.S., Musheshe N. and Schmidt M. (2019) Function of cAMP scaffolds in obstructive lung disease: focus on epithelial-to-mesenchymal transition and oxidative stress. Br. J. Pharmacol. 176, 2402–2415 10.1111/bph.14605 30714124 PMC6592852

[58] Oldenburger A., Timens W., Bos S., Smit M., Smrcka A.V., Laurent A.-C. et al. (2014) Epac1 and Epac2 are differentially involved in inflammatory and remodeling processes induced by cigarette smoke. FASEB J. 28, 4617–4628 10.1096/fj.13-248930 25103224 PMC4200332

[59] Laudette M., Zuo H., Lezoualc'h F. and Schmidt M. (2018) Epac function and cAMP scaffolds in the heart and lung. J. Cardiovasc. Dev. Dis. 5, 9 10.3390/jcdd5010009 PMC5872357 29401660

[60] Roscioni S.S., Prins A.G., Elzinga C.R., Menzen M.H., Dekkers B.G., Halayko A.J. et al. (2011) Protein kinase A and the exchange protein directly activated by cAMP (Epac) modulate phenotype plasticity in human airway smooth muscle. Br. J. Pharmacol. 164, 958–969 10.1111/j.1476-5381.2011.01354.x 21426315 PMC3195918

[61] Laurent A.C., Bisserier M., Lucas A., Tortosa F., Roumieux M., De Regibus A. et al. (2015) Exchange protein directly activated by cAMP 1 promotes autophagy during cardiomyocyte hypertrophy. Cardiovasc. Res. 105, 55–64 10.1093/cvr/cvu242 25411381

[62] Prajapati R., Fujita T., Suita K., Nakamura T., Cai W., Hidaka Y. et al. (2019) Usefulness of exchanged protein directly activated by cAMP (Epac)1-inhibiting therapy for prevention of atrial and ventricular arrhythmias in mice. Circ. J. 83, 295–303 10.1253/circj.CJ-18-0743 30518738

[63] Zhang L., Zhang L., Liu H., Jiang F., Wang H., Li D. et al. (2018) Inhibition of Epac2 attenuates neural cell apoptosis and improves neurological deficits in a rat model of traumatic brain injury. Front. Neurosci. 12, 263 10.3389/fnins.2018.00263 29740274 PMC5924794

[64] Zhuang Y., Xu H., Richard S.A., Cao J., Li H., Shen H. et al. (2019) Inhibition of EPAC2 attenuates intracerebral hemorrhage-induced secondary brain injury via the p38/BIM/Caspase-3 pathway. J. Mol. Neurosci. 67, 353–363 10.1007/s12031-018-1215-y 30607901

[65] Almahariq M., Tsalkova T., Mei F.C., Chen H., Zhou J., Sastry S.K. et al. (2013) A novel EPAC-specific inhibitor suppresses pancreatic cancer cell migration and invasion. Mol. Pharmacol. 83, 122–128 10.1124/mol.112.080689 23066090 PMC3533471

[66] Zhu Y., Chen H., Boulton S., Mei F., Ye N., Melacini G. et al. (2015) Biochemical and pharmacological characterizations of ESI-09 based EPAC inhibitors: defining the ESI-09 “therapeutic window”. Sci. Rep. 5, 9344 10.1038/srep09344 25791905 PMC4366844

[67] Enserink J.M., Christensen A.E., de Rooij J., van Triest M., Schwede F., Genieser H.G. et al. (2002) A novel Epac-specific cAMP analogue demonstrates independent regulation of Rap1 and ERK. Nat. Cell Biol. 4, 901–906 10.1038/ncb874 12402047

[68] Su Y., Dostmann W.R., Herberg F.W., Durick K., Xuong N.H., Ten Eyck L. et al. (1995) Regulatory subunit of protein kinase A: structure of deletion mutant with cAMP binding domains. Science 269, 807–813 10.1126/science.7638597 7638597

[69] Schwede F., Bertinetti D., Langerijs C.N., Hadders M.A., Wienk H., Ellenbroek J.H. et al. (2015) Structure-guided design of selective Epac1 and Epac2 agonists. PLoS Biol. 13, e1002038 10.1371/journal.pbio.1002038 25603503 PMC4300089

[70] Christensen A.E., Selheim F., de Rooij J., Dremier S., Schwede F., Dao K.K. et al. (2003) cAMP analog mapping of Epac1 and cAMP kinase. Discriminating analogs demonstrate that Epac and cAMP kinase act synergistically to promote PC-12 cell neurite extension. J. Biol. Chem. 278, 35394–35402 10.1074/jbc.M302179200 12819211

[71] Vliem M.J., Ponsioen B., Schwede F., Pannekoek W.-J., Riedl J., Kooistra M.R.H. et al. (2008) 8-pCPT-2′-O-Me-cAMP-AM: an improved Epac-selective cAMP analogue. ChemBioChem 9, 2052–2054 10.1002/cbic.200800216 18633951

[72] Yang N.J. and Hinner M.J. (2015) Getting across the cell membrane: an overview for small molecules, peptides, and proteins In Site-Specific Protein Labeling: Methods and Protocols (Gautier A. and Hinner M. J. eds), pp. 29-53, Springer New York, New York, NY 10.1007/978-1-4939-2272-7_3 PMC4891184 25560066

[73] Schultz C., Vajanaphanich M., Harootunian A.T., Sammak P.J., Barrett K.E. and Tsien R.Y. (1993) Acetoxymethyl esters of phosphates, enhancement of the permeability and potency of cAMP. J. Biol. Chem. 268, 6316–6322 PMID:8384207

[74] Kang G., Joseph J.W., Chepurny O.G., Monaco M., Wheeler M.B., Bos J.L. et al. (2003) Epac-selective cAMP analog 8-pCPT-2′-*O*-Me-cAMP as a stimulus for Ca^2+^-induced Ca^2+^ release and exocytosis in pancreatic beta-cells. J. Biol. Chem. 278, 8279–8285 10.1074/jbc.M211682200 12496249 PMC3516291

[75] Kooistra M.R., Corada M., Dejana E. and Bos J.L. (2005) Epac1 regulates integrity of endothelial cell junctions through VE-cadherin. FEBS Lett. 579, 4966–4972 10.1016/j.febslet.2005.07.080 16115630

[76] Fukuhara S., Sakurai A., Sano H., Yamagishi A., Somekawa S., Takakura N. et al. (2005) Cyclic AMP potentiates vascular endothelial cadherin-mediated cell-cell contact to enhance endothelial barrier function through an Epac–Rap1 signaling pathway. Mol. Cell. Biol. 25, 136–146 10.1128/MCB.25.1.136-146.2005 15601837 PMC538793

[77] Sands W.A., Woolson H.D., Milne G.R., Rutherford C. and Palmer T.M. (2006) Exchange protein activated by cyclic AMP (Epac)-mediated induction of suppressor of cytokine signaling 3 (SOCS-3) in vascular endothelial cells. Mol. Cell. Biol. 26, 6333–6346 10.1128/MCB.00207-06 16914720 PMC1592846

[78] Leech C.A., Dzhura I., Chepurny O.G., Schwede F., Genieser H.-G. and Holz G.G. (2010) Facilitation of β-cell K_ATP_ channel sulfonylurea sensitivity by a cAMP analog selective for the cAMP-regulated guanine nucleotide exchange factor Epac. Islets 2, 72–81 10.4161/isl.2.2.10582 20428467 PMC2860288

[79] Pannekoek W.-J., Vliem M.J. and Bos J.L. (2018) Multiple Rap1 effectors control Epac1-mediated tightening of endothelial junctions. Small GTPases, 1–8 10.1080/21541248.2018.1431512 PMC7549671 29388865

[80] Fazal L., Laudette M., Paula-Gomes S., Pons S., Conte C., Tortosa F. et al. (2017) Multifunctional mitochondrial Epac1 controls myocardial cell death. Circ. Res. 120, 645–657 10.1161/CIRCRESAHA.116.309859 28096195

[81] Gu Y., Li G. and Huang L.-Y.M. (2018) Inflammation induces Epac-protein kinase C alpha and epsilon signaling in TRPV1-mediated hyperalgesia. Pain 159, 2383–2393 10.1097/j.pain.0000000000001346 30015706

[82] Ebrahimighaei R., McNeill M.C., Smith S.A., Wray J.P., Ford K.L., Newby A.C. et al. (2019) Elevated cyclic-AMP represses expression of exchange protein activated by cAMP (EPAC1) by inhibiting YAP-TEAD activity and HDAC-mediated histone deacetylation. Biochim. Biophys. Acta Mol. Cell Res. 1866, 1634–1649 10.1016/j.bbamcr.2019.06.013 31255721

[83] Stokman G., Qin Y., Genieser H.-G., Schwede F., de Heer E., Bos J.L. et al. (2011) Epac-Rap signaling reduces cellular stress and ischemia-induced kidney failure. J. Am. Soc. Nephrol. 22, 859–872 10.1681/ASN.2010040423 21493776 PMC3083308

[84] Stokman G., Qin Y., Booij T.H., Ramaiahgari S., Lacombe M., Dolman M.E.M. et al. (2014) Epac-Rap signaling reduces oxidative stress in the tubular epithelium. J. Am. Soc. Nephrol. 25, 1474–1485 10.1681/ASN.2013070679 24511123 PMC4073429

[85] Hothi S.S., Gurung I.S., Heathcote J.C., Zhang Y., Booth S.W., Skepper J.N. et al. (2008) Epac activation, altered calcium homeostasis and ventricular arrhythmogenesis in the murine heart. Pflugers Arch. 457, 253–270 10.1007/s00424-008-0508-3 18600344 PMC3714550

[86] Pereira L., Cheng H., Lao D.H., Na L., van Oort R.J., Brown J.H. et al. (2013) Epac2 mediates cardiac β 1-adrenergic-dependent sarcoplasmic reticulum Ca^2+^ leak and arrhythmia. Circulation 127, 913–922 10.1161/CIRCULATIONAHA.12.148619 23363625 PMC3690126

[87] Hwang M., Go Y., Park J.-H., Shin S.-K., Song S.E., Oh B.-C. et al. (2017) Epac2a-null mice exhibit obesity-prone nature more susceptible to leptin resistance. Int. J. Obes. (Lond) 41, 279–288 10.1038/ijo.2016.208 27867203 PMC5309344

[88] Zhang C.-L., Katoh M., Shibasaki T., Minami K., Sunaga Y., Takahashi H. et al. (2009) The cAMP sensor Epac2 is a direct target of antidiabetic sulfonylurea drugs. Science 325, 607–610 10.1126/science.1172256 19644119

[89] Herbst K.J., Coltharp C., Amzel L.M. and Zhang J. (2011) Direct activation of Epac by sulfonylurea is isoform selective. Chem 18, 243–251 10.1016/j.chembiol.2010.12.007 PMC3130306 21338921

[90] Tsalkova T., Blumenthal D.K., Mei F.C., White M.A. and Cheng X. (2009) Mechanism of Epac activation: structural and functional analyses of Epac2 hinge mutants with constructive and reduced activities. J. Biol. Chem. 284, 23644–23651 10.1074/jbc.M109.024950 19553663 PMC2749139

[91] Tsalkova T., Gribenko A.V. and Cheng X. (2011) Exchange protein directly activated by cyclic AMP isoform 2 is not a direct target of sulfonylurea drugs. Assay Drug Dev. Technol. 9, 88–91 10.1089/adt.2010.0338 21133673 PMC3033205

[92] Charles M.A., Lawecki J., Steiner A.L. and Grodsky G.M. (1976) Cyclic nucleotides in pancreatic islets: tolbutamide- and arginine-induced insulin release. Diabetes 25, 256–259 10.2337/diab.25.4.256 178555

[93] Grill V. (1977) Cyclic amp and insulin release. Acta Paediatr. 66, 41–47 10.1111/j.1651-2227.1977.tb15120.x 210617

[94] Guček A., Gandasi N.R., Omar-Hmeadi M., Bakke M., Døskeland S.O., Tengholm A. et al. (2019) Fusion pore regulation by cAMP/Epac2 controls cargo release during insulin exocytosis. eLife 8, e41711 10.7554/eLife.41711 31099751 PMC6557626

[95] Ammazzalorso A., De Filippis B., Giampietro L. and Amoroso R. (2017) *N*-acylsulfonamides: synthetic routes and biological potential in medicinal chemistry. Chem. Biol. Drug Des. 90, 1094–1105 10.1111/cbdd.13043 28632928

[96] Carnaroglio D., Martina K., Palmisano G., Penoni A., Domini C. and Cravotto G. (2013) One-pot sequential synthesis of isocyanates and urea derivatives via a microwave-assisted Staudinger–aza-Wittig reaction. Beilstein J. Org. Chem. 9, 2378–2386 10.3762/bjoc.9.274 24367403 PMC3869261

[97] Tanwar D.K., Ratan A. and Gill M.S. (2017) A facile synthesis of sulfonylureas via water assisted preparation of carbamates. Org. Biomol. Chem. 15, 4992–4999 10.1039/C7OB00872D 28567464

[98] Kreye O., Mutlu H. and Meier M.A.R. (2013) Sustainable routes to polyurethane precursors. Green Chem. 15, 1431–1455 10.1039/c3gc40440d

[99] Parnell E., McElroy S.P., Wiejak J., Baillie G.L., Porter A., Adams D.R. et al. (2017) Identification of a novel, small molecule partial agonist for the cyclic AMP sensor, EPAC1. Sci. Rep. 7, 294 10.1038/s41598-017-00455-7 28331191 PMC5428521

[100] Kageyama K., Tamasawa N. and Suda T. (2011) Signal transduction in the hypothalamic corticotropin-releasing factor system and its clinical implications. Stress 14, 357–367 10.3109/10253890.2010.536279 21438777

[101] Zhu J., Mix E. and Winblad B. (2001) The antidepressant and antiinflammatory effects of rolipram in the central nervous system. CNS Drug Rev. 7, 387–398 10.1111/j.1527-3458.2001.tb00206.x 11830756 PMC6741679

[102] Wei Z., Jiang W., Wang H., Li H., Tang B., Liu B. et al. (2018) The IL-6/STAT3 pathway regulates adhesion molecules and cytoskeleton of endothelial cells in thromboangiitis obliterans. Cell. Signal. 44, 118–126 10.1016/j.cellsig.2018.01.015 29339086

[103] Drelich A., Judy B., He X., Chang Q., Yu S., Li X. et al. (2018) Exchange protein directly activated by cAMP modulates ebola virus uptake into vascular endothelial cells. Viruses 10, 563 10.3390/v10100563 PMC6213290 30332733

[104] Ballatore C., Huryn D.M. and Smith A.B. III (2013) Carboxylic acid (bio)isosteres in drug design. ChemMedChem 8, 385–395 10.1002/cmdc.201200585 23361977 PMC3640829

[105] Meanwell N.A. (2011) Synopsis of some recent tactical application of bioisosteres in drug design. J. Med. Chem. 54, 2529–2591 10.1021/jm1013693 21413808

[106] Enyeart J.A. and Enyeart J.J. (2009) Metabolites of an Epac-selective cAMP analog induce cortisol synthesis by adrenocortical cells through a cAMP-independent pathway. PloS One 4, e6088 10.1371/journal.pone.0006088 19564912 PMC2698983

[107] Herfindal L., Krakstad C., Myhren L., Hagland H., Kopperud R., Teigen K. et al. (2014) Introduction of aromatic ring-containing substituents in cyclic nucleotides is associated with inhibition of toxin uptake by the hepatocyte transporters OATP 1B1 and 1B3. PloS One 9, e94926 10.1371/journal.pone.0094926 24740327 PMC3989234

[108] Herfindal L., Nygaard G., Kopperud R., Krakstad C., Doskeland S.O. and Selheim F. (2013) Off-target effect of the Epac agonist 8-pCPT-2′-*O*-Me-cAMP on P2Y12 receptors in blood platelets. Biochem. Biophys. Res. Commun. 437, 603–608 10.1016/j.bbrc.2013.07.007 23850619

[109] Poppe H., Rybalkin S.D., Rehmann H., Hinds T.R., Tang X.B., Christensen A.E. et al. (2008) Cyclic nucleotide analogs as probes of signaling pathways. Nat. Methods 5, 277–278 10.1038/nmeth0408-277 18376388

[110] Sand C., Grandoch M., Borgermann C., Oude Weernink P.A., Mahlke Y., Schwindenhammer B. et al. (2010) 8-pCPT-conjugated cyclic AMP analogs exert thromboxane receptor antagonistic properties. Thromb. Haemost. 103, 662–676 10.1160/TH09-06-0341 20135060

[111] Nenquin M. and Henquin J.C. (2016) Sulphonylurea receptor-1, sulphonylureas and amplification of insulin secretion by Epac activation in β cells. Diabetes Obes. Metab. 18, 698–701 10.1111/dom.12607 26584950

[112] Barella L.F., Rossi M., Zhu L., Cui Y., Mei F.C., Cheng X. et al. (2019) Beta-cell-intrinsic beta-arrestin 1 signaling enhances sulfonylurea-induced insulin secretion. J. Clin. Invest. 130, 3732–3737 10.1172/JCI126309 31184597 PMC6715363

